# Interaction of Dihydrocitrinone with Native and Chemically Modified Cyclodextrins

**DOI:** 10.3390/molecules24071328

**Published:** 2019-04-04

**Authors:** Zelma Faisal, Sándor Kunsági-Máté, Beáta Lemli, Lajos Szente, Dominik Bergmann, Hans-Ulrich Humpf, Miklós Poór

**Affiliations:** 1 Department of Pharmacology, Faculty of Pharmacy, University of Pécs, Szigeti út 12, 7624 Pécs, Hungary; faisal.zelma@gytk.pte.hu; 2 János Szentágothai Research Center, University of Pécs, Ifjúság útja 20, 7624 Pécs, Hungary; kunsagi-mate.sandor@gytk.pte.hu (S.K.-M.); beata.lemli@aok.pte.hu (B.L.); 3 Department of Pharmaceutical Chemistry, Faculty of Pharmacy, University of Pécs, Rókus u. 2, 7624 Pécs, Hungary; 4 Institute of Organic and Medicinal Chemistry, Medical School, University of Pécs, Szigeti út 12, H-7624 Pécs, Hungary; 5 CycloLab Cyclodextrin Research & Development Laboratory, Ltd., Illatos út 7, 1097 Budapest, Hungary; szente@cylolab.hu; 6 Institute of Food Chemistry, Westfälische Wilhelms-Universität Münster, Corrensstr. 45, 48149 Münster, Germany; d.bergmann@uni-muenster.de (D.B.); humpf@wwu.de (H.-U.H.)

**Keywords:** dihydrocitrinone, cyclodextrin, citrinin, fluorescence spectroscopy, host-guest interaction

## Abstract

Citrinin (CIT) is a nephrotoxic mycotoxin produced by *Aspergillus*, *Penicillium*, and *Monascus* genera. It appears as a contaminant in grains, fruits, and spices. After oral exposure to CIT, its major urinary metabolite, dihydrocitrinone (DHC) is formed, which can be detected in human urine and blood samples. Cyclodextrins (CDs) are ring-shaped molecules built up from glucose units. CDs can form host-guest type complexes with several compounds, including mycotoxins. In this study, the complex formation of DHC with native and chemically modified beta- and gamma-cyclodextrins was tested at a wide pH range, employing steady-state fluorescence spectroscopic and modeling studies. The weakly acidic environment favors the formation of DHC-CD complexes. Among the CDs tested, the quaternary-ammonium-γ-cyclodextrin (QAGCD) formed the most stable complexes with DHC. However, the quaternary-ammonium-β-cyclodextrin (QABCD) induced the strongest enhancement in the fluorescence signal of DHC. Our results show that some of the chemically modified CDs are able to form stable complexes with DHC (log*K* = 3.2–3.4) and the complex formation can produce even a 20-fold increase in the fluorescence signal of DHC. Considering the above-listed observations, CD technology may be a promising tool to increase the sensitivity of the fluorescence detection of DHC.

## 1. Introduction

**Figure 1:**
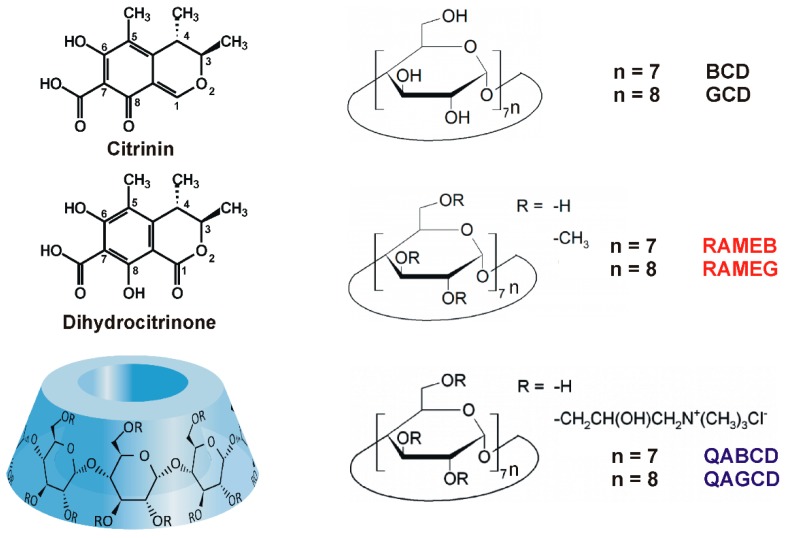
Chemical structures of citrinin, dihydrocitrinone, randomly-substituted methyl cyclodextrins (degree of substitution: 12), and randomly-substituted quaternary ammonium cyclodextrins (degree of substitution: 3–4 and 3–4.5 for RAMEB and RAMEG, respectively) tested.

Citrinin (CIT) is a nephrotoxic mycotoxin produced by *Penicillium*, *Aspergillus* and *Monascus* species [1]. It occurs as a contaminant in cereals, spices, fruits, as well as in Asian foods and beverages (e.g., cheese, sake, and miso). Furthermore, CIT appears in red mold rice used as a red pigment in the Asian food industry [2,3]. Dry heating of CIT at 175 °C results in the formation of its nontoxic degradation products, and in moist conditions, the toxicity of CIT decreases with the increase of heat [4]. However, at 140–160 °C, similarly toxic products (as the parent compound) are produced. Therefore, the elimination of CIT from the food chain is difficult [4]. After oral exposure, CIT is extensively metabolized and its main urinary metabolite dihydrocitrinone (DHC; Figure 1) is formed [5,6]. Because DHC is a less toxic compound than CIT, it is a detoxification process [7,8]. Based on previous reports, DHC appears in detectable quantities in human blood and urine samples [6,9,10,11].

Cyclodextrins (CD) are starch-derived molecules built up from D-glucopyranose units. The most frequently applied CDs are α-, β-, and γ-cyclodextrins containing six, seven, and eight glucose units, respectively (Figure 1). The lipophilic internal cavity of CDs is able to accommodate apolar guest molecules, while their hydrophilic external part makes CDs strongly hydrophilic [12,13]. Chemical modification of CDs highly affects the stability of the formed host-guest type complexes as well as the selectivity of the CDs towards the guest molecule [12]. There is an increasing trend of applying CDs in the pharmaceutical, cosmetic, and food industries [14]. Previous studies highlighted that CDs form stable complexes with several mycotoxins, including ochratoxin A, aflatoxins, citrinin, and zearalenone, and zearalenols [15,16,17,18,19,20,21,22]. As a result of the complex formation, CDs can strongly increase the fluorescence signal of fluorophores, therefore, the analytical application of CDs to enhance the fluorescence detection of some mycotoxins seems a promising field [13,23,24,25]. Previous studies also revealed that CD technology is suitable to extract mycotoxins from aqueous solutions (including beverages) for analytical or decontamination purposes [26,27,28]. Because CIT can interact with CDs [19,29], it is reasonable to hypothesize that DHC is also able to form stable complexes with native or chemically modified CDs.

In this study, we aimed to investigate the complex formation of DHC with native and chemically modified cyclodextrins at a wide pH range, employing steady-state fluorescence spectroscopic and modeling studies. CD-induced enhancement in the fluorescence signal of DHC was tested, and the stability of formed DHC-CD complexes was evaluated. Finally, modeling studies were performed for deeper understanding of DHC-CD interactions. Our results demonstrate that some of the chemically-modified CDs are able to form stable complexes with DHC and the complex formation can produce a strong increase in the fluorescence signal of DHC.

## 2. Results and Discussion

### 2.1. Fluorescence Properties of DHC and CIT in Different Buffers

**Figure 2:**
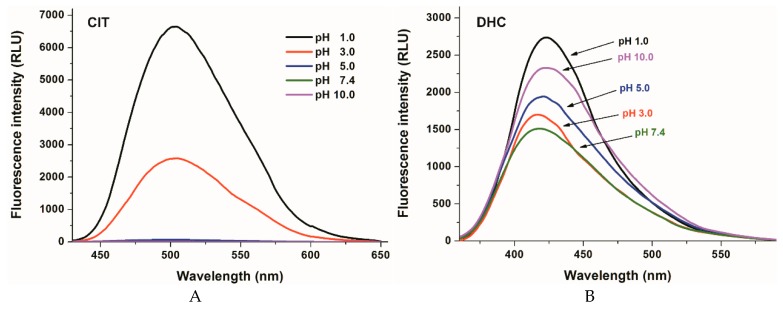
Fluorescence emission spectra of CIT (**A**; 10 µM; λ_ex_ = 330 nm) and DHC (**B**; 10 µM; λ_ex_ = 325 nm) in different buffers (pH 1.0: 0.10 M hydrogen chloride; pH 3.0: 0.05 M sodium citrate buffer; pH 5.0: 0.05 M sodium acetate buffer; pH 7.4: 0.05 M sodium phosphate buffer; pH 10.0: 0.05 M sodium borate buffer).

To test the effect of environmental pH on the fluorescence signals of DHC and CIT, their fluorescence emission spectra were recorded in different buffers (pH 1.0–10.0, see details in 3.1; Figure 2). CIT exerts fluorescence emission signal only at strongly acidic conditions, while at pH 5 its fluorescence signal almost completely disappears (λ_ex_ = 330 nm, Figure 2A). The pKa value of CIT is approximately 3.5 [30]. Since the ionized form of the mycotoxin does not exert significant fluorescence, the fluorescence signal of CIT gradually disappears with the increase of the pH [29]. However, DHC showed fluorescence property at the whole pH range tested (λ_ex_ = 325 nm; Figure 2B). The complex changes in the fluorescence signal of DHC in HCl and different buffers may be derived partly from the deprotonation of the carboxyl and/or phenolic hydroxyl groups. Nevertheless, other environmental conditions (e.g., buffer components and ionic strength) are also able to affect the fluorescence signal of DHC (as it is demonstrated in Figure S1).

### 2.2. Effects of Native α-, β-, and γ-CDs on the Fluorescence Signal of DHC

**Figure 3:**
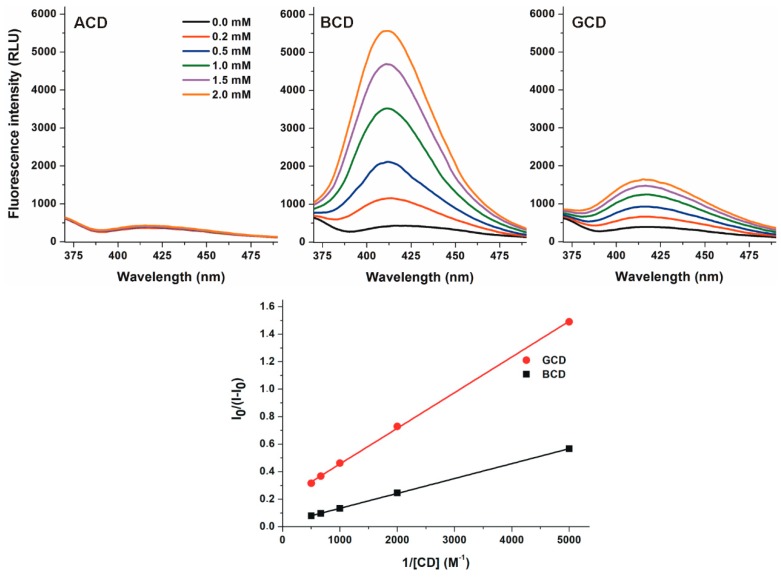
**Top**: Fluorescence emission spectrum of DHC (2 µM) in the presence of increasing concentrations of α-, β-, and γ-CD (0.0–2.0 mM; λ_ex_ = 325 nm, λ_em_ = 410 nm) in 0.05 M sodium acetate buffer (pH 5.0). **Bottom**: Benesi-Hildebrand plots of DHC-BCD and DHC-GCD complexes.

To investigate the interactions of native α-(ACD), β-(BCD), and γ-CDs (GCD) with DHC, increasing amounts of CDs (final concentrations: 0.0–2.0 mM) were added to DHC (2 µM) in 0.05 M sodium acetate buffer (pH 5.0), then fluorescence emission spectra were recorded (λ_ex_ = 325 nm). ACD did not modify the emission spectrum of DHC. However, BCD and GCD strongly increased the fluorescence of the mycotoxin (Figure 3). Because the applied CDs do not exert fluorescence, the CD-induced enhancement in the fluorescence signal of DHC needs to be resulted from the formation of DHC-CD complexes. The microenvironment in the CD cavity is less polar than in water, thus the fluorescence signal of DHC is strongly increased by the apolar microenvironment. The entrapment of the fluorophore in the CD cavity leads to the partial decomposition of its hydration shell and consequently decreases the quenching effect of water molecules. Based on these principles, the significant increase of the fluorescence emission signal of a fluorophore can be resulted from the host-guest type complex formation with CDs [15,22,23,24]. Furthermore, the DHC molecule likely has a tighter skeleton in the complex with BCD compared to the DHC-GCD complex, which may also be an explanation regarding the higher fluorescence in the presence of BCD vs. GCD. These results suggest that DHC does not form complexes with ACD (likely because of the small diameter of the CD cavity), while both BCD and GCD are able to interact with the mycotoxin. These results are consistent with the previous investigation with CIT: ACD did not affect the fluorescence of CIT while BCD induced a stronger increase in the fluorescence of the mycotoxin than GCD (at pH 2.0) [29]. Furthermore, CDs caused only 1.3–2.1-fold increases in the fluorescence of CIT (at pH 2.0). However, even the native BCD induced more than ten-fold increase in the fluorescence signal of DHC (at pH 5.0). Despite the interaction of DHC with BCD resulted in stronger enhancement in its fluorescence compared to GCD (Figure 3, top), the binding constant of DHC-GCD proved to be higher than the DHC-BCD complex (see later in Table 1 and Table 2), as it is also demonstrated by the Benesi–Hildebrand plots (Figure 3, bottom). In contrast, CIT forms more stable complexes with BCD (the binding constant is approximately three-fold higher) than with GCD [29].

### 2.3. Interaction of DHC with β-Cyclodextrins

**Figure 4:**
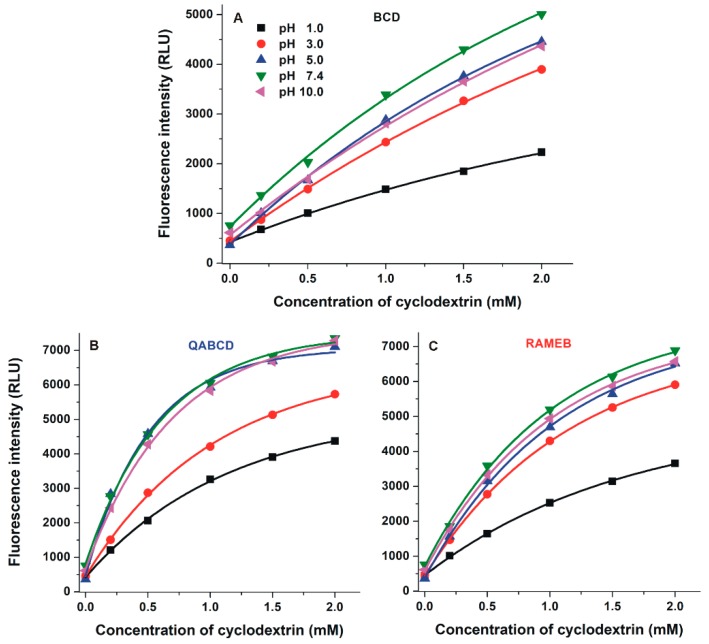
Enhancement in the fluorescence signal of DHC (2 µM) by increasing concentrations of β-CDs (**A**: BCD, **B**: QABCD, and **C**: RAMEB; 0.0–2.0 mM) in different buffers (λ_ex_ = 325 nm, λ_em_ = 410 nm; ex slit: 10 nm, em slit: 20 nm; pH 1.0: 0.10 M hydrogen chloride; pH 3.0: 0.05 M sodium tartrate buffer; pH 5.0: 0.05 M sodium acetate buffer; pH 7.4: 0.05 M TRIS-HCl buffer; pH 10.0: 0.05 M sodium borate buffer).

Since the fluorescence spectrum of DHC highly depends on the environmental conditions, the complex formation of DHC with CDs was tested at a wide pH range (pH 1.0–10.0; see details in 3.1). Fluorescence emission spectrum of DHC (2 µM) was recorded in the presence of increasing β-CD concentrations (0.0–2.0 mM). Regardless of the buffer used, each β-CD induced strong enhancement in the fluorescence of DHC (Figure 4 and Figure S2); therefore, the complex formation of DHC with CDs seems significant in a wide pH range.

The weakest β-CD-induced (BCD, QABCD, and RAMEB) increase in the fluorescence of DHC was observed at pH 1.0 and 3.0 (Figure 4). However, weakly acidic and alkaline environments (pH 5.0, 7.4, and 10.0) favor the formation of highly fluorescent complexes, which may result from the ionization of the mycotoxin. DHC-QABCD and DHC-RAMEB complexes showed higher absolute fluorescence than the DHC-BCD complex (Figure 4). Since the fluorescence signal of DHC is also strongly affected by the pH (see Figure 2B), the relative changes in its fluorescence (I/I_0_) show different tendencies. Typically, the lowest relative enhancement in the fluorescence of DHC was observed at pH 1.0 while the highest was produced at pH 5.0 (Table 1). Depending on the circumstances, QABCD, RAMEB, and BCD induced 10–20-fold, 7–18-fold, and 5–13-fold relative increase in the fluorescence of DHC, respectively. Because CIT loses its fluorescence at higher pH (see Figure 2A), effects of CDs on the fluorescence signal of CIT were investigated at pH 2.0 in our previous study [29]. BCD and RAMEB caused only 1.7- and 2.0-fold increases in the fluorescence of CIT, respectively.

**Figure 5:**
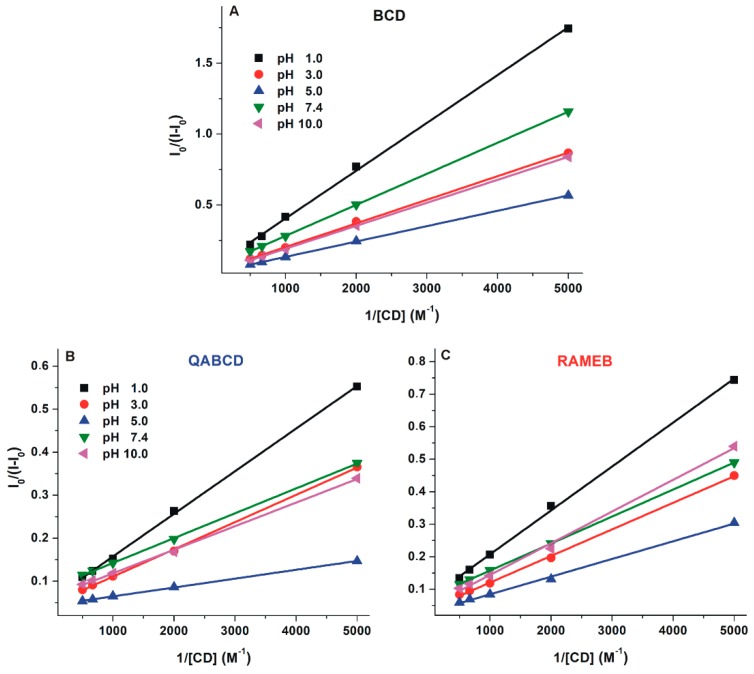
Benesi–Hildebrand plots of DHC-β-CD complexes (**A**: BCD, **B**: QABCD, and **C**: RAMEB) in different buffers (λ_ex_ = 325 nm, λ_em_ = 410 nm; pH 1.0: 0.10 M hydrogen chloride; pH 3.0: 0.05 M sodium tartrate buffer; pH 5.0: 0.05 M sodium acetate buffer; pH 7.4: 0.05 M TRIS-HCl buffer; pH 10.0: 0.05 M sodium borate buffer).

Binding constants (*K*; unit: L/mol) of DHC-β-CD complexes were determined by employing the graphical application of the Benesi-Hildebrand equation (Equation (1)). Experimental data showed excellent linearity with the 1:1 stoichiometry model (Figure 5) and suggest the formation of DHC-β-CD complexes with log*K* values in the 2.2–3.2 range (Table 1). Binding constants of DHC-β-CD complexes were similar to CIT-β-CD (log*K* = 2.3–2.9) [29]. Among β-CDs, the native BCD formed the less stable complexes with DHC, and similarly to the DHC-RAMEB complex, only slight pH-dependent changes in the complex stability were observed. However, the stability of DHC-QABCD complex significantly increased with the increase of the pH, showing a maximum at pH 5.0 and 7.4, after which a slight decrease was noticed at pH 10.0 (Table 1). At pH 5.0 and 7.4, the binding constants of DHC-QABCD complexes are approximately two- and six-fold higher compared to DHC-RAMEB and DHC-BCD, respectively. The improved binding ability of QABCD under weakly acidic and neutral conditions likely results from the deprotonation of the carboxyl group of DHC. The formation of the DHC anion may interact with the cationic part of the QABCD molecule, therefore, the ionic interactions can further stabilize the inclusion. The slight decrease in the stability of DHC-QABCD complex at pH 10.0 may have resulted from the deprotonation of the phenolic hydroxyl group(s), which can likely influence the complex formation. From this point of view, DHC behaves differently from CIT: CIT-β-CD complexes are more stable at lower pH [29].

### 2.4. Interaction of DHC with γ-Cyclodextrins

**Figure 6:**
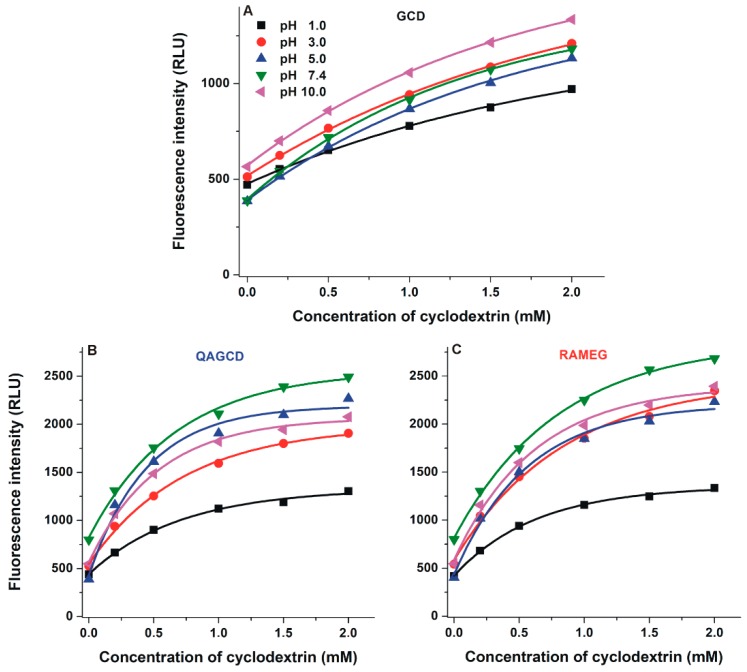
Enhancement in the fluorescence signal of DHC (2 µM) by increasing concentrations of γ-CDs (**A**: GCD, **B**: QAGCD, and **C**: RAMEG; 0.0–2.0 mM) in different buffers (λ_ex_ = 325 nm, λ_em_ = 410 nm; ex slit: 10 nm, em slit: 20 nm; pH 1.0: 0.10 M hydrogen chloride; pH 3.0: 0.05 M sodium tartrate buffer; pH 5.0: 0.05 M sodium acetate buffer; pH 7.4: 0.05 M TRIS-HCl buffer; pH 10.0: 0.05 M sodium borate buffer).

Despite BCD induced significantly higher enhancement in the fluorescence of DHC (see Figure 3), DHC forms more stable complexes with GCD (Table 1 and Table 2). Therefore, the interaction of DHC with GCD as well as its methyl (RAMEG) and quaternary ammonium (QAGCD) derivatives was also investigated. The fluorescence emission spectrum of DHC (2 µM) was recorded in the presence of increasing concentrations of γ-CDs (0.0–2.0 mM) in different buffers (pH 1.0–10.0; see details in Section 3.1). The tested γ-CDs are non-fluorescent molecules. However, each γ-CD increased significantly the fluorescence signal of DHC, suggesting the formation of DHC-γ-CD complexes (Figure 6). Interestingly, the stability of DHC-GCD complex is almost ten-fold higher compared with CIT-GCD [29].

The weakest increase in the fluorescence of DHC was observed at pH 1.0 regarding each γ-CD tested. The strongest absolute fluorescence intensities were detected in the presence of QAGCD and RAMEG at pH 7.4 (Figure 6), showing that chemically modified γ-CDs are able to induce considerably larger enhancement in the fluorescence of the mycotoxin compared to the native GCD. Since the fluorescence signal of DHC is highly influenced by the pH, the relative changes in its fluorescence (I/I_0_) need to be separately evaluated. The highest increase in the relative fluorescence was observed at pH 5.0 with each γ-CD tested (Table 2). From this point of view, QAGCD was the strongest enhancer, followed by RAMEG and GCD.

**Figure 7:**
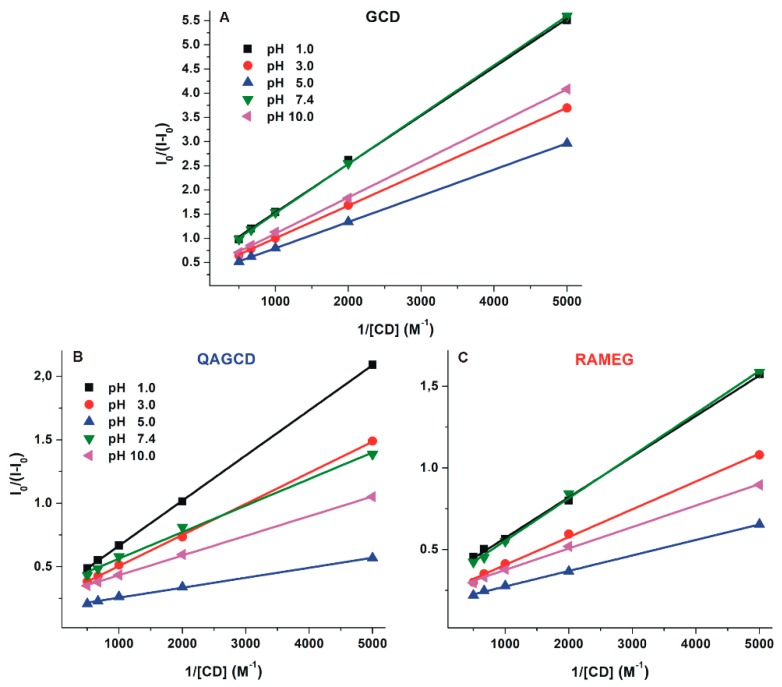
Benesi–Hildebrand plots of DHC-γ-CD complexes (**A**: GCD, **B**: QAGCD, and **C**: RAMEG) in different buffers (λ_ex_ = 325 nm, λ_em_ = 410 nm; pH 1.0: 0.10 M hydrogen chloride; pH 3.0: 0.05 M sodium tartrate buffer; pH 5.0: 0.05 M sodium acetate buffer; pH 7.4: 0.05 M TRIS-HCl buffer; pH 10.0: 0.05 M sodium borate buffer).

Binding constants of DHC-γ-CD complexes were determined by the Benesi–Hildebrand equation (Equation (1)). Benesi–Hildebrand plots are demonstrated in Figure 7. Experimental data show excellent linearity with the 1:1 stoichiometry model, suggesting the formation of complexes with log*K* values in the 2.7–3.4 range (Table 2). Among γ-CDs tested, GCD formed the less stable complexes with DHC. Furthermore, only slight pH-dependent changes in the complex stability of DHC-GCD and DHC-RAMEG complexes were observed. However, the stability of the DHC-QAGCD complex significantly increases with the increase of the pH, showing a maximum at pH 5.0, after which a slight decrease was noticed at pH 7.4 and 10.0 (Table 2). As we discussed in Section 2.3, the observed changes in the stability of DHC-QAGCD complex likely result from the ionization of the mycotoxin.

### 2.5. Modeling Studies

Calculations were performed in the absence and presence of water molecules. Table 3 (top) summarizes the thermodynamic parameters of the complex formation of DHC with BCD or GCD host molecules calculated at semi-empirical AM1 level and considering the differently charged ionic states of DHC. The results suggest significantly different mechanisms regarding DHC-BCD and DHC-GCD complex formations. However, complex stabilities are similar at room temperature. The larger cavity of the GCD molecule offers more space for water molecules remaining in the solvation shell of DHC. This property is enhanced at elevated pH because of the stronger Coulomb interactions between the negatively charged DHC and the solvent water molecules. The lower enhancement in the fluorescence signals of DHC by GCD vs. BCD also supports this hypothesis.

Considering the known quenching effect of water on the fluorescence signal of molecules possessing aromatic moieties [31] as well as our experimental findings described in 2.2–2.4, the increase in the fluorescence signal of DHC in the presence of CD molecules highlights the removal of water quencher solvent molecules from the solvation shell of the DHC guest, due to the lipophilic interior of CD molecules. This property is supported by modeling studies: the dehydrated DHC molecule interacts with the lipophilic cavity of BCD or GCD derivatives. Although the enhancement of the fluorescence signal of DHC is proportional to the pH, there is no further enhancement in the fluorescence signals above pH 5 (Table 1 and Table 2). The correlation can be obtained between the complex stabilities and the enhancement in the fluorescence signals. However, above pH 5, this correlation is not so clear. Molecular modeling studies suggest the following hypothesis: the ionization of DHC molecules supports the better stabilization of water molecules in their hydration shell, therefore, at least one water molecule will still coordinate to the DHC anion during and after its inclusion by the CD cavity. This property causes two competitive effects according to the changes in the fluorescence signal: the remained water molecule quenches partly the fluorescence of DHC (decrease the fluorescence intensity), while the rigid molecular skeleton (caused by the secondary bonds with participation of water molecules) does not support collisions with the neighboring solvent molecules (increase the fluorescence intensity). The latest process also causes enhancement in the fluorescence signal. Modeling results show that DHC molecule prefers to enter into the BCD cavity by its methyl-terminated end. However, regarding the ionized forms of DHC (especially in the case of quaternary-ammonium derivative), the permanent dipole moment of the DHC interacts with the positively charged outer regions of the QABCD molecule. As a result, the charged (−1 or −2 electronic charge) DHC molecule enters into the cavity of QABCD by its deprotonated carboxyl moiety. This conformation retains one water molecule in the solvation shell of DHC, causing much weaker enhancement in the entropy term associated with the complex formation.

## 3. Materials and Methods 

### 3.1. Reagents

The synthesis of (±)-dihydrocitrinone (DHC) was performed based on the synthetic procedure for (±)-[^13^C_3_]-dihydrocitrinone reported by Bergmann et al. [32]. Stock solution of DHC (5000 µM) was prepared in ethanol (96 *v*/*v*%, Reanal, spectroscopic grade) and stored protected from light at −20°C. Cyclodextrins, including α-cyclodextrin (ACD), β-cyclodextrin (BCD), γ-cyclodextrin (GCD), randomly methylated β-cyclodextrin (RAMEB), (2-hydroxy-3-*N*,*N*,*N*-trimethylamino)propyl-β-cyclodextrin chloride (QABCD), randomly methylated γ-cyclodextrin (RAMEG), and (2-hydroxy-3-*N*,*N*,*N*-trimethylamino)propyl-γ-cyclodextrin chloride (QAGCD) were provided by CycloLab Cyclodextrin Research and Development Laboratory, Ltd. Hydrogen chloride (HCl, 0.10 M, pH 1.0), sodium tartrate buffer (0.05 M, pH 3.0), sodium citrate buffer (0.05 M, pH 3.0), sodium phosphate buffer (0.05 M, pH 3.0), sodium acetate buffer (0.05 M, pH 5.0), TRIS-HCl buffer (0.05 M, pH 7.4), sodium phosphate buffer (0.05 M, pH 7.4), Na-HEPES buffer (0.05 M, pH 7.4), and sodium borate buffer (0.05 M, pH 10.0) were prepared and used as media during spectroscopic measurements.

### 3.2. Fluorescence Spectroscopic Measurements

Steady-state fluorescence measurements were carried out using a Hitachi F-4500 fluorimeter. The experiments were performed in a wide pH range (1.0–10.0; see details in 3.1). All analyses were carried out in the presence of air at +25 °C. Fluorescence spectra were recorded in the presence of DHC (2 μM) and increasing concentrations of CDs (0.0, 0.2, 0.5, 1.0, 1.5, and 2.0 mM), employing 325 and 410 nm as excitation and emission wavelengths, respectively.

Binding constants (*K*) of DHC-CD complexes were determined by the graphical application of the Benesi-Hildebrand equation, assuming 1:1 stoichiometry [19]:

(1) $$\frac{I_{0}}{\left(I-I_{0}\right)}=\frac{1}{A}+\frac{1}{A\times K\times {\left[H\right]}^{n}}$$

where *I* is the fluorescence intensity of DHC in the presence of CD, *I_0_* is the fluorescence intensity of DHC in the absence of CD, *[H]* stands for the concentration of CDs, *A* is a constant, *n* is the number of binding sites, and *K* (unit: L/mol) denotes the binding constant.

### 3.3. Modeling Studies

Molecular modeling studies have been performed at semi-empirical AM1 level using HyperChem 8 code. The enthalpy change of the complex formation was considered as the energy change calculated by subtracting the total energies of the reactants from the total energies of the products. To consider the overall effect of the entropy changes, the different terms of the entropy contents of all species were calculated applying the Boltzmann statistics. For example, after calculating the vibrational frequencies using the harmonic approximation, the entropy was calculated on the common way using the following HyperChem code:

(2) $$S_{vib}=R\sum_{i}\{\frac{h\nu_{i}/kT}{e^{\left(h\nu_{i}/kT\right)}-1}-\ln[1-e^{\left(-h\nu_{i}/kT\right)}]\}$$

where *ν_i_* is the frequency of vibration and *T* is the temperature (298.16 K). The entropy change associated with the complex formation was then determined by subtracting the entropy content of the reactants from the total entropy content of the product.

## 4. Conclusions

In summary, the interaction of DHC with native and chemically modified CDs was investigated under different environmental conditions, applying steady-state fluorescence spectroscopic and molecular modeling studies. Formation of DHC-CD complexes (log*K* = 2.2–3.4) was observed with β- and γ-CDs (BCD, QABCD, RAMEB, GCD, QAGCD, RAMEG). Chemical modification of native CDs can result in the significantly stronger enhancement in the fluorescence of the mycotoxin as well as the formation of more stable DHC-CD complexes. β-CDs induced considerably stronger enhancement in the fluorescence emission signal of DHC than γ-CDs. However, γ-CDs form more stable complexes with the mycotoxin. The highest absolute fluorescence of DHC was noticed in the presence of QABCD at pH range 5.0–10.0, while the most stable complex was DHC-QAGCD at pH 5.0. The positive charge of quaternary ammonium CDs can enhance the complex formation, likely through the stabilization of the mycotoxin-CD complex with ionic interaction. Based on our observations, the analytical utilization of the DHC-CD complex formation seems promising.

## Figures and Tables

**Table 1:** Decimal logarithmic values of binding constants (*K*; unit: L/mol) of DHC-β-CD (BCD, QABCD, and RAMEB) complexes in different buffers and β-CD-induced relative increase in the fluorescence of DHC (I/I_0_) (2 μM DHC + 2 mM β-CD; λ_ex_ = 325 nm, λ_em_ = 410 nm; pH 1.0: 0.10 M hydrogen chloride; pH 3.0: 0.05 M sodium tartrate buffer; pH 5.0: 0.05 M sodium acetate buffer; pH 7.4: 0.05 M TRIS-HCl buffer; pH 10.0: 0.05 M sodium borate buffer; SEM, standard error of mean).

pH	DHC–BCD		DHC–QABCD		DHC–RAMEB	
	log*K* ± SEM	I/I_0_ ± SEM	log*K* ± SEM	I/I_0_ ± SEM	log*K* ± SEM	I/I_0_ ± SEM
1.0	2.29 ± 0.04	5.59 ± 0.12	2.76 ± 0.01	10.17 ± 0.11	2.76 ± 0.02	6.98 ± 0.11
3.0	2.34 ± 0.04	9.23 ± 0.35	2.81 ± 0.02	12.89 ± 0.18	2.79 ± 0.04	12.89 ± 0.19
5.0	2.47 ± 0.05	13.14 ± 0.63	3.23 ± 0.05	19.53 ± 0.06	2.78 ± 0.02	17.59 ± 0.12
7.4	2.42 ± 0.06	6.75 ± 0.06	3.21 ± 0.02	9.92 ± 0.07	2.90 ± 0.03	9.31 ± 0.14
10.0	2.22 ± 0.02	7.40 ± 0.36	3.03 ± 0.02	14.22 ± 0.34	2.85 ± 0.04	12.74 ± 0.34

**Table 2:** Decimal logarithmic values of binding constants (*K*; unit: L/mol) of DHC-γ-CD (GCD, QAGCD, and RAMEG) complexes in different buffers, and γ-CD-induced relative increase in the fluorescence of DHC (I/I_0_) (2 μM DHC + 2 mM CD; λ_ex_ = 325 nm, λ_em_ = 410 nm; pH 1.0: 0.10 M hydrogen chloride; pH 3.0: 0.05 M sodium tartrate buffer; pH 5.0: 0.05 M sodium acetate buffer; pH 7.4: 0.05 M TRIS-HCl buffer; pH 10.0: 0.05 M sodium borate buffer; SEM, standard error of mean).

pH	DHC–GCD		DHC–QAGCD		DHC–RAMEG	
	log*K* ± SEM	I/I_0_ ± SEM	log*K* ± SEM	I/I_0_ ± SEM	log*K* ± SEM	I/I_0_ ± SEM
1.0	2.69 ± 0.03	2.09 ± 0.03	2.98 ± 0.03	2.96 ± 0.06	3.12 ± 0.01	3.12 ± 0.08
3.0	2.78 ± 0.05	2.41 ± 0.10	3.13 ± 0.05	3.70 ± 0.12	3.20 ± 0.06	4.21 ± 0.09
5.0	2.73 ± 0.04	3.05 ± 0.07	3.38 ± 0.04	6.01 ± 0.09	3.22 ± 0.03	5.64 ± 0.08
7.4	2.75 ± 0.03	2.00 ± 0.01	3.18 ± 0.02	3.48 ± 0.16	3.05 ± 0.02	3.46 ± 0.05
10.0	2.74 ± 0.06	2.32 ± 0.07	3.24 ± 0.02	3.83 ± 0.02	3.18 ± 0.05	4.42 ± 0.03

**Table 3:** Thermodynamic parameters of the complex formation of DHC with BCD or GCD, RAMEB, and QABCD. Calculations performed at semi-empirical AM1 level.

**Ionic State of DHC**	**DHC–BCD**				**DHC–GCD**			
	**log*K***	**Δ*G***	**Δ*H***	**Δ*S***	**log*K***	**Δ*G***	**Δ*H***	**Δ*S***
0	2.3	−18.65	−14.60	13.6	2.7	−21.90	−22.85	−3.19
−1	2.5	−20.28	−16.02	14.3	2.7	−21.94	−26.45	−15.13
−2	2.2	−17.84	−13.19	15.6	2.7	−21.97	−28.19	−20.86
	**DHC–RAMEB**				**DHC–QABCD**			
	**log*K***	**Δ*G***	**Δ*H***	**Δ*S***	**log*K***	**Δ*G***	**Δ*H***	**Δ*S***
0	2.7	−21.90	−17.85	13.6	2.7	−21.90	−17.85	13.6
−1	2.8	−22.71	−18.45	14.3	3.3	−26.76	−25.07	5.7
−2	2.8	−22.71	−18.06	15.6	3.0	−24.33	−22.49	6.2

## Supplementary Materials

The following are available: Figure S1: Fluorescence emission spectrum of DHC (10 µM; λex = 325 nm) in different buffers at pH 3.0 (left) and pH 7.4 (right). Figure S2: Representative fluorescence emission spectra of DHC (2 µM) in the presence of increasing concentrations of QABCD (0.0–2.0 mM) in different buffers (λex = 325 nm; ex slit: 10 nm, em slit: 10 nm; pH 1.0: 0.10 M hydrogen chloride; pH 3.0: 0.05 M sodium tartrate buffer; pH 5.0: 0.05 M sodium acetate buffer; pH 7.4: 0.05 M TRIS-HCl buffer; pH 10.0: 0.05 M sodium borate buffer).
