# Supplementary material for: Interaction of Dihydrocitrinone with Native and Chemically Modified Cyclodextrins
Source: Molecules. 2019 Apr 4;24(7):1328. doi: 10.3390/molecules24071328 (PMC6479545; doi:10.3390/molecules24071328)
Supplement: Supplementary file 1 [file molecules-24-01328-s001.pdf]

molecules MDPI

Article

Interaction of dihydrocitrinone with native and chemically modified cyclodextrins

SUPPLEMENTARY MATERIALS

Zelma Faisal [1,2], Sándor Kunsági-Máté [2,3], Beáta Lemli [2,4], Lajos Szente [5], Dominik Bergmann [6], Hans-Ulrich Humpf [6], and Miklós Poór [1,2,*]

[1] Department of Pharmacology, University of Pécs, Faculty of Pharmacy, Szigeti út 12, Pécs 7624, Hungary; faisal.zelma@gytk.pte.hu
[2] János Szentágothai Research Center, University of Pécs, Ifjúság útja 20, Pécs 7624, Hungary; kunsagi-mate.sandor@gytk.pte.hu (S.K.), beata.lemli@aok.pte.hu (B.L.)
[3] Department of Pharmaceutical Chemistry, University of Pécs, Faculty of Pharmacy, Rókus u. 2, Pécs 7624, Hungary
[4] Institute of Organic and Medicinal Chemistry, Medical School, University of Pécs, Szigeti út 12, H-7624 Pécs, Hungary
[5] CycloLab Cyclodextrin Research & Development Laboratory, Ltd., Illatos út 7, Budapest 1097, Hungary; szente@cylolab.hu
[6] Institute of Food Chemistry, Westfälische Wilhelms-Universität Münster, Corrensstr. 45, 48149 Münster, Germany; d.bergmann@uni-muenster.de (D.B.), humpf@wwu.de (H.-U.H.)

* Correspondence: poor.miklos@pte.hu; Tel.: +36-72-536-000 Ext: 35052 (M.P.)

Received: date; Accepted: date; Published: date

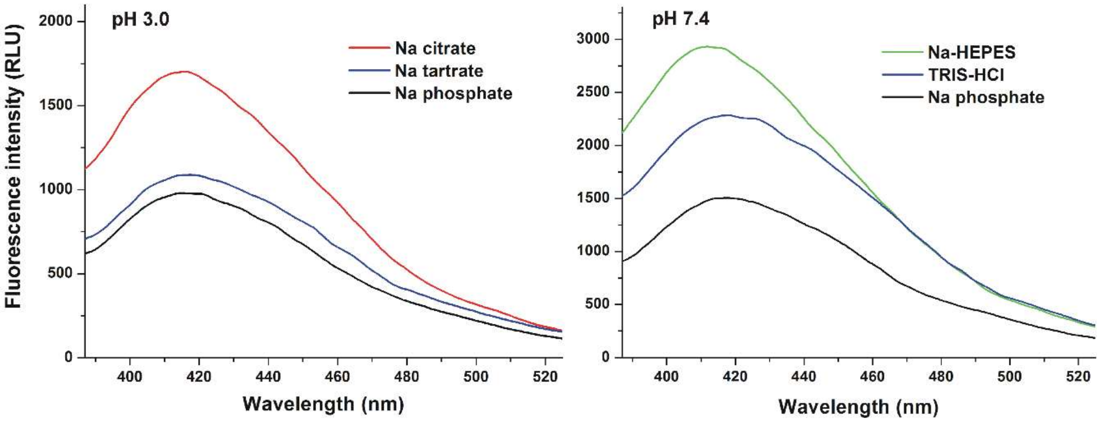

Figure S1. Fluorescence emission spectrum of DHC (10 μM; λ_{ex} = 325 nm) in different buffers at pH 3.0 (left) and pH 7.4 (right).

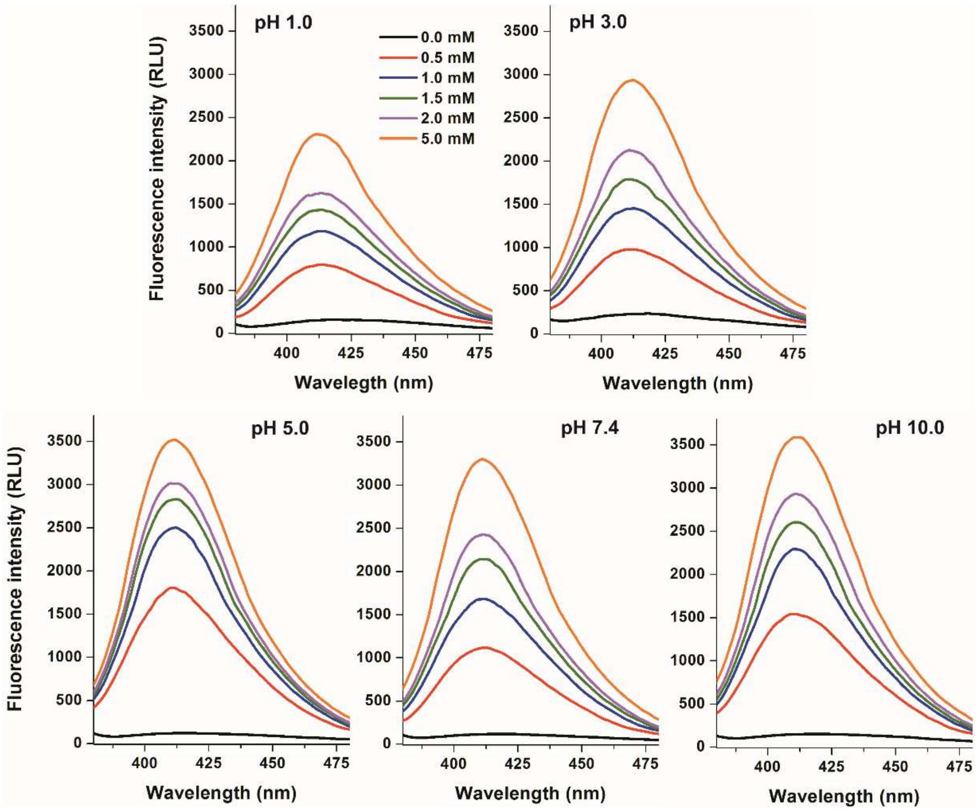

Figure S2. Representative fluorescence emission spectra of DHC (2 μM) in the presence of increasing concentrations of QABCD (0.0-2.0 mM) in different buffers (λ_{ex} = 325 nm; ex slit: 10 nm, em slit: 10 nm; pH 1.0: 0.10 M hydrogen chloride; pH 3.0: 0.05 M sodium tartrate buffer; pH 5.0: 0.05 M sodium acetate buffer; pH 7.4: 0.05 M TRIS-HCl buffer; pH 10.0: 0.05 M sodium borate buffer).
